# Outcome Predictors of Acute-on-Chronic Liver Failure: A Narrative Review

**DOI:** 10.7759/cureus.61655

**Published:** 2024-06-04

**Authors:** Nitish Batra, Shilpa A Gaidhane, Sunil Kumar, Sourya Acharya

**Affiliations:** 1 Medicine, Jawaharlal Nehru Medical College, Datta Meghe Institute of Higher Education and Research, Wardha, IND

**Keywords:** organ failure, model for end stage liver disease (meld), clif-c-aclf score, prognostic markers, acute on chronic liver failure

## Abstract

Complications of acute-on-chronic liver failure (ACLF) include increased short-term mortality. Extrahepatic organ failures result from chronic liver disease and acute hepatic injury. This combination characterizes end-stage liver disease. Its rapid progression makes it challenging for hepatologists and intensivists to treat. The varied definitions of this condition lead to varied clinical presentations. Hepatic or extrahepatic failures are more prevalent in chronic hepatitis B or cirrhosis patients who receive an additional injury. Numerous intensity parameters and prognosis ratings, including those for hepatitis B virus (HBV), have been developed and verified for various patients and causes of the disease. Liver regeneration, liver transplantation (LT), or antiviral therapy for HBV-related ACLF are the main treatment aims for various organ failures. LT is the best treatment for HBV-ACLF. In some HBV-related ACLF patients, nucleos(t)ide analogs and artificial liver assistance may enhance survival. Combining epidemiological and clinical studies, this review updates our understanding of HBV-ACLF's definition, diagnosis, epidemiology, etiology, therapy, and prognosis.

## Introduction and background

Both acute-on-chronic liver failure (ACLF) and acute decompensation of liver cirrhosis are significant but pathophysiologically different clinical entities observed in patients referred to hospitals with cirrhosis-related complications [1]. The final phase, cirrhosis, is marked by the emergence of regenerating nodules and a persistent, chronic inflammatory response. It is also accompanied by increasing fibrosis [2]. Once cirrhosis has taken hold, it cannot be reversed and is linked to higher morbidity, the emergence of potentially fatal complications, and a worse standard of living. However, ACLF is a distinct clinical illness defined by extrahepatic or hepatic organ failure, with a considerable 28-day death rate of more than 15%. Three primary characteristics define this syndrome: infections or alcoholic hepatitis are examples of pro-inflammatory triggering events that are closely linked to the development of ACLF. Additionally, ACLF is related to single- or multiple-organ failure and occurs in an environment of elevated systemic inflammation [3]. In chronic liver illness, ACLF is characterized by a sharp decline in liver function and is frequently linked to the quick emergence of life-threatening side effects such as hepatic encephalopathy (HE) and hepatorenal syndrome [4]. With 28- and 90-day fatalities exceeding 25% and 40%, respectively, ACLF has a significant rate of short-term mortality [5]. For certain patients, liver transplantation (LT) can be the only effective course of treatment. The Model for End-stage Liver Disease (MELD) and Model for End-stage Liver Disease-Sodium (MELD-Na) scores are two predictive scores that aid in directing the distribution of donor livers for transplantation. However, these scoring systems lack crucial factors that represent the functional and nutritional state of patients with ACLF. Extensive research has shown that the ACLF severity among candidates selected for LT was not captured by the MELD score [6].

A 'compensated' or asymptomatic phase is followed by a rapidly progressive phase known as 'decompensated' in the clinical course of the disease. This causes the two primary cirrhosis-related syndromes, portal hypertension as well as hepatic insufficiency, to manifest as consequences (jaundice, variceal bleeding, ascites, and encephalopathy) [7]. As a result, cirrhosis has four stages: two compensated stages (non-hemorrhagic esophageal varices or absence of ascites) and two decompensated phases (variceal bleeding and presence of ascites). These stages are associated with portal pressure gradient changes and fibrosis histological stages [8]. Sepsis, or infection in patients with critical illnesses, is thought to represent the fifth stage, and its existence has been linked to a higher death rate [9].

Hepatic decompensation may occur after acute injury in cirrhosis patients with extensive fibrosis and decreased functional reserve. Clinical practice suggests two decompensation techniques for these individuals. The first and most prevalent kind is called chronic liver decompensation, which progresses during the period of end-stage liver disease. The second type is called acute liver decompensation, which happens suddenly as a consequence of an acute insult, such as sepsis, alcoholism, or variceal bleeding in patients who have already had compensated liver disease. ACLF will be used to describe this condition, which is characterized by a significant death rate ranging between 50% and 90% in the short- or medium-term and rapid advancement that calls for support for multiple organ failure. It is critical to distinguish between chronic decompensation caused by chronic liver disease development, usually permanent, and ACLF, which can be reversed if the triggering reason is alleviated [10]. When two 'lesions' or 'insults,' one chronic and the other acute, occur on the liver simultaneously, the condition was initially referred to as ACLF in 1995 [11]. The definition put forth by the American Association for the Study of Liver Diseases (AASLD) and the European Association for the Study of the Liver (EASL) is as follows: the acute exacerbation of preexisting chronic liver disease, typically triggered by a precipitating event, which is accompanied by a heightened risk of mortality within three months due to the failure of multiple organ systems [12].

## Review

Search methodology 

**Figure 1:**
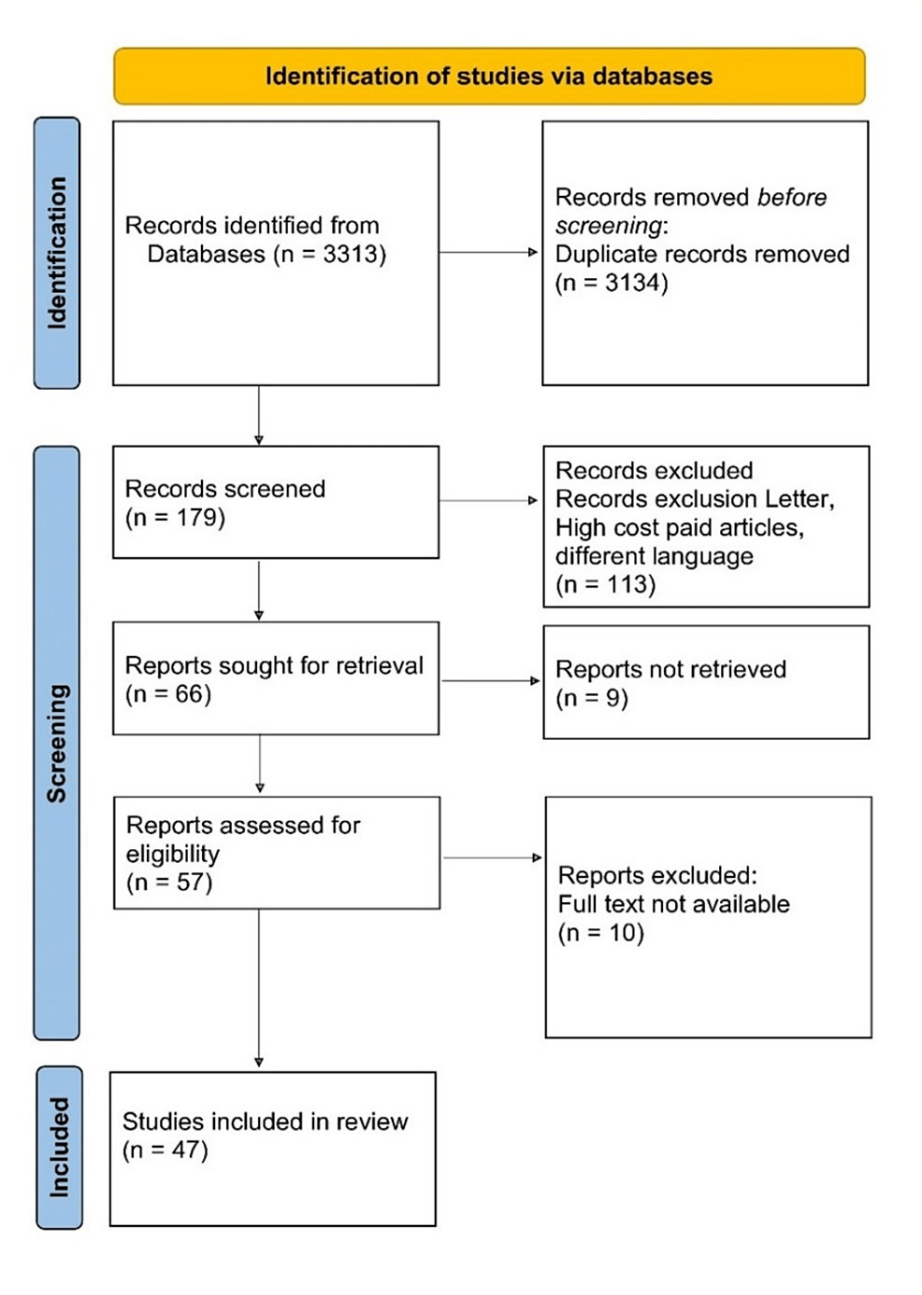
The selection process of articles used in this study Adopted from the Preferred Reporting Items for Systemic Reviews and Meta-Analyses (PRISMA)

A comprehensive literature search was conducted using electronic databases such as PubMed, Medical Literature Analysis and Retrieval System Online (MEDLINE), PsycINFO, and Cochrane Library. The search encompassed articles published from 2000 to the present. The search was conducted using Medical Subject Heading (MeSH) terms. It utilized specific keywords such as 'acute on chronic liver failure,' 'model for end-stage liver disease (meld),' 'CLIF-C ACLF score,' 'prognostic markers,' and 'organ failure.' Articles published between 2000 and 2023 were included in the review. After the initial search, articles were screened based on their relevance to the topic and inclusion criteria. The inclusion criteria included articles that evaluated prevention strategies for adolescent drug use and overdose, provided data on the effectiveness of prevention strategies, and identified risk factors for overdose deaths. The inclusion criteria needed them all to be present in a selected article. The exclusion criteria included articles that focused solely on adult populations, were not published in English, or were not peer-reviewed. Figure 1 describes the selection process of articles used in our study.

Pathophysiology

**Figure 2:**
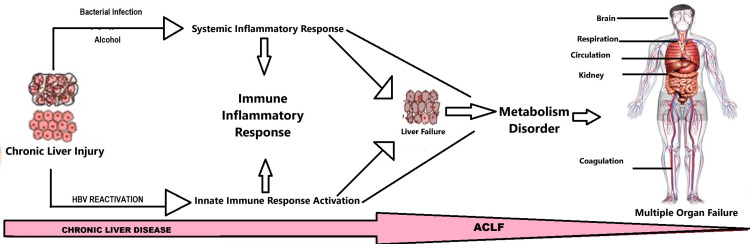
Pathophysiology of ACLF ACLF: acute-on-chronic liver failure; HBV: hepatitis B virus The image is created by the corresponding author

ACLF develops with compensated cirrhosis and a proinflammatory cytokine-rich systemic inflammatory response. This cytokine profile may mediate liver cell inflammation, apoptosis, necrosis, fibrosis, and cholestasis [13]. Patients diagnosed with ALD and ACLF exhibit elevated levels of inflammatory response markers, including white blood cell count and C-reactive protein (CRP), which are strongly associated with prognosis. The concentrations of proinflammatory cytokines, namely interleukin (IL)-6 and IL-8, as well as anti-inflammatory cytokine IL-10, are markedly elevated in the bloodstream of these individuals [14]. Pathogen-associated molecular patterns (PAMPs) and damage-associated molecular patterns (DAMPs) can cause systemic inflammation. The leading cause of ACLF is bacterial infection, which can result in elevated levels of PAMPs in the blood that are detectable by pattern-recognition receptors (PRRs). Engaging PRR can activate intracellular signaling cascades, triggering the development of a cytokine storm, an imbalance in pro- and anti-inflammatory regulation, and the transcription and synthesis of inflammatory mediators [15]. Virulence factors comprise the second category of inflammatory agents produced by microorganisms. These factors generally elude the detection of specialized receptors; instead, they are discerned via the consequences of their activity, a mechanism known as functional feature recognition. Complementary immune responses are believed to be elicited at the site of infection in response to the identification of bacteria by recognizing their structural and functional characteristics. These responses are designed to eradicate the invading pathogen [16]. Sterile inflammation refers to systemic inflammation in the absence of infection. When many DAMPs are released from damaged or dead hepatocytes, certain PRRs are triggered. This produces proinflammatory cytokines, creating an inflammatory cascade and leading to acute coronary syndrome [17]. High mobility group box 1 (HMGB1) production by oxidatively challenged and hypoxic hepatocytes indicates ischemia-reperfusion liver damage [18], as shown in Figure 2.

During tissue hypoperfusion, PAMPs and mediators of inflammation increase splanchnic arteriolar wall inducible nitric oxide (NO) synthase. The overproduction of NO produces splanchnic vasodilation and decreased effective arterial blood volume, amplifying the endogenous neurohumoral vasoconstrictor network homeostatically. Neurohumoral mediators cause renal vessels to contract, causing acute kidney injury, glomerular filtration rate reduction, and kidney hypoperfusion [19,20]. Immune metabolism disorder: ACLF and immunodeficiency are commonly seen together. By lowering the quantity of Kupffer cells, innate-immune protein molecules, and PRRs, liver cirrhosis, which is characterized by the replacement of the sound endothelial reticulum architecture of the liver alongside fibrous septa and liver fibers, significantly raises the likelihood of bacterial infection, systemic inflammatory response, and sepsis [21]. Blood leukocytosis, or activated white blood cells that can invade tissues and cause immunopathology akin to sepsis seen in the general population, is often associated with ACLF [20]. About cirrhosis, there is certain proof in support of this theory. Tumor necrosis factor-α and NF-κB-dependent signaling pathways, for instance, could be involved in hepatocyte death, NO-mediated pulmonary dysfunction, and macrophage accumulation in the lung microvasculature [22]. Many patients with acutely decompensated cirrhosis (consortium acute-on-chronic liver failure in cirrhosis (CANONIC) cohort) demonstrated decreased mitochondrial fatty acid β-oxidation in peripheral organs during high-throughput blood metabolomics. It would reduce oxidative phosphorylation and build adenosine triphosphate (ATP). These findings suggest ACLF organ failures may arise due to poor energy generation [23]. Hepatitis B e-antigen and other hepatitis B virus (HBV) proteins can interfere with Kupffer cells' capacity to express toll-like receptors, prevent some T lymphocytes from increasing, and prevent them from secreting IL-10 and interferon-γ [24]. Decompensated cirrhosis and ACLF neutrophils created more neutrophil extracellular traps but had worse phagocytosis, oxygen-reactive production, and bactericidal activity [25]. 

Patients with ACLF exhibit markedly reduced monocyte-macrophage system activity, including reduced proinflammatory factor release, bacterial killing, oxidative burst, and phagocytosis [26]. The proportion of CD163^+^/CD206^+^ macrophages rises as HBV-related liver disorders worsen, and macrophage polarization gradually shifts from classically to alternatively activated. Patients with ACLF have increased myeloid-derived suppressor cells, which inhibit T-cell activity to reduce antimicrobial innate immune responses [27]. Interferon, monocyte, neutrophil, and dendritic cell gene modules were significantly upregulated during the disease's progression from chronic hepatitis B or liver cirrhosis toward ACLF T cell, B cell, and natural killer cell gene modules were significantly downregulated. The primary mechanisms of HBV-ACLF are immunological dysregulation brought on by HBV reactivation, which includes innate immune system activation and adaptive immune system fatigue [28]. The primary metabolic organ of the body, the liver, also processes amino acids, stores glucose, and maintains the equilibrium of lipids and cholesterol. New insights into the biology of chronic liver disease have been brought about by recent developments showing predominate metabolic changes in the course of ACLF illness. Elevated levels of high-density lipoprotein cholesterol and altered serum lysophosphatidylcholine, two metabolic dysregulations that indicate hepatocyte death, are linked to an elevated severity as well as mortality of decompensated liver disease. Throughout all phases of ACLF development, significant changes to metabolic pathways such as lipid metabolism, autophagy, and oxygen homeostasis were seen, highlighting the significance of immune-metabolism dysfunction as a possible mechanism of ACLF [28,29]. In the leukocytes, mitochondrial malfunction controls immunometabolism, and bioenergetic failure represents a new element in the pathogenesis [30]. Systemic inflammatory processes, mitochondrial dysfunction, and sympathetic nervous system activation are good short-term death predictors [31]. 

Management

**Figure 3:**
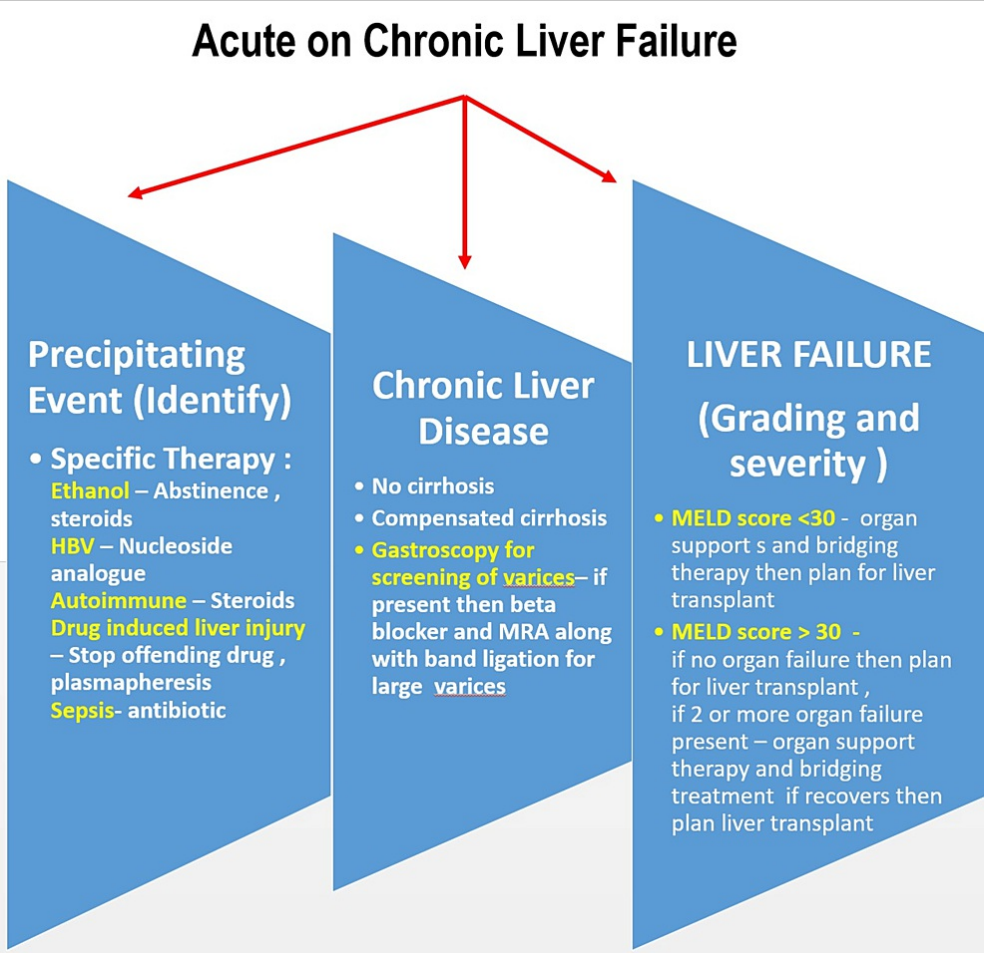
Schematic flow chart showing an overview of disease management HBV: hepatitis B virus; MELD: model for end-stage liver disease; MRA: mineralocorticoid receptor antagonist The image is created by the corresponding author

As of present, there is no specific treatment for ACLF. The two primary tenets of treatment are supportive therapy and the diagnosis and management of triggering events. Patients with life-threatening single- or multi-organ failure, unresponsive to routine medication, may benefit from organ support in an intensive care unit, under the supervision of specialists who specialize in the management of liver disease. A schematic flowchart showing an overview of disease management is shown in Figure 3.

Three laboratory variables are included in the MELD score, the most widely utilized framework for predicting the prognosis of liver disease: creatinine, international normalized ratio (INR), and total bilirubin (TBIL). Originally designed to forecast survival in patients receiving elective transjugular intrahepatic portosystemic shunt (TIPSS) installation due to problems from portal hypertension, the MELD was developed. Following validation, the MELD, which solely relies on objective variables, was a reliable indicator of survival across various patient groups with advanced liver disorder. The allocation of organs for LT has been the primary application of the MELD score. The MELD score can also predict survival in cirrhosis patients with infections, variceal bleeding, fulminant hepatic failure, and alcoholic hepatitis. MELD can help pick the optimum treatment for hepatocellular carcinoma patients who aren't eligible for LT and select patients for other surgeries. Despite its benefits, the MELD score cannot predict survival for 15% to 20% of patients. The model's prediction power may improve by adding factors indicating liver and renal function. The MELD score is constantly improved and confirmed [32]. The MELD score considers renal insufficiency and hepatic dysfunction; however, it leaves out other important conditions that can impact prognosis like infection, organ failure, or HE [33]. In patients with ACLF, scores apart from MELD or MELD-Na, depending on organ failure, might be a better indicator of prognosis. 

Numerous characteristics, such as age, AFP, as well as platelets, have been linked in the past to poor outcomes associated with liver failure [34]. In individuals with ACLF, the frequency of infection, whether aggravating or precipitating, is more than 50% and increases with the severity of the condition. While bacteria are typically the causing organisms, fungal infections are also not unheard of. The suspected site should determine the earliest possible start of antimicrobial treatment, the culture and isolation results, and the area's antimicrobial sensitivity patterns [35].

The most recent society guidelines (Baveno/EASL) for treating a variceal hemorrhage episode should be followed. Volume restoration, a safe vasoconstrictor drug combination, antibacterial prevention, and ideally, endoscopic therapy performed within 12 hours of presentation, are all part of the usual medical treatment. A patient who exhibits haematemesis should also get an elective intubation. Preventive TIPSS and rescue TIPSS increase survival in patients with ACLF. According to two additional investigations, the presence of ACLF is an important indicator of death in individuals with variceal bleeding [36].

Even though prednisolone medication is recommended for patients with alcoholic hepatitis, prednisolone responsiveness is inversely correlated with the number of organ failures at baseline. Steroid use in ACLF patients is made more difficult by their increased susceptibility to new infections. A thorough evaluation of persistent infection is crucial when choosing a steroid treatment. On day 7, the Lille score should be used to evaluate the steroid response; if there is no improvement by then, the medication should be stopped [37].

Except for hepatitis B, there is no particular treatment for acute viral hepatitis. Strong nucleotide or nucleoside analogs must be started as soon as possible if the patient has an HBV infection at diagnosis, pending confirmation based on the viral deoxyribonucleic acid burden. Western organ failure is most often caused by acute renal injury. Therefore, urine testing to determine acute tubular necrosis or type 1 hepatorenal syndrome, diuretic withdrawal, and intravenous albumin volume expansion should be done [38]. Vasoconstrictor agent treatment should be initiated if diuretics are stopped and the volume is expanded without any improvement. The number of organ failures is inversely correlated with the probability of a renal response to vasoconstriction. The first-choice vasopressor for treating prolonged shock is norepinephrine [39]. Elective intubation should protect the airway when a patient has high-grade encephalopathy (grade 3 and grade 4). HE can resolve quickly with lactulose treatment and concurrent use of rifaximin; caution should be used to avoid causing excessive diarrhea. Patients having grade 3 or grade 4 HE, who are not responding to lactulose treatment, may benefit from albumin dialysis. Extracorporeal liver support device randomized clinical trials have not yet demonstrated a benefit in mortality for individuals with ACLF. In contrast to conventional medical care, these include albumin dialysis and an extracorporeal liver device incorporating hepatocytes [40].

Currently, the only therapeutic option for ACLF patients is LT. Patients with a few organ failures with ACLF do not significantly vary from those without organ failures in terms of their one-year survival rates following LT. Patients who experience three or more organ failures have a post-transplant survival rate of about 80%, while patients who do not receive an LT have a survival rate of less than 20% [41]. One successful treatment for ACLF is LT. An international joint study in Europe revealed that 234 ACLF patients had an 81% one-year post-LT survival rate, demonstrating the efficacy of LT for survival [42]. However, it is unclear how LT should be prioritized in these populations. Currently, the MELD score is used globally for organ allocation. However, research has revealed that because the MELD score does not account for extrahepatic organ failure, it isn't responsive or adequately precise for prognosis prediction in ACLF. The prognosis and the best time to initiate long-term care (LT) for patients with ACLF should be evaluated using the criteria and predictive scores, like the Chinese Group on the Study of Severe Hepatitis B (COSSH)-ACLF score and the Chronic Liver Failure Consortium (CLIF-C) ACLF score, created especially for these patients. To address these problems, European researchers have suggested that revised organ allocation guidelines for individuals with ACLF be adopted globally and have developed a global study known as the CHANCE trial [42,43]. Optimizing candidates for LT listings and transplantation may also benefit from dynamic evaluation of the disease severity following hospitalization. The CANONIC trial found that ACLF-2 and ACLF-3 patients were at high risk of early mortality, requiring LT. Patients having four or more organ failures or a CLIF-C ACLF score > 64 on days 3-7 may be considered for LT futility if they have other LT contraindications. To lower the risk of ineffective LT, the COSSH Project now evaluates the outcomes of HBV-ACLF patients who have undergone LT and suggests a net survival benefit-based priority for HBV-ACLF LT. The COSSH-ACLF II score accurately predicted survival benefits, post-LT mortality, and waitlist death in HBV-ACLF patients. LT recipients with a 7-10 COSSH-ACLF II score had better net survival [43]. Our understanding of how ACLF patients may progress from chronic liver failure to death has increased, but many areas still need additional research. In the upcoming years, it is anticipated that ACLF will become more common due to the rising frequency of associated risk factors for the emergence of chronic liver disease, which include drug misuse, alcoholism, and obesity [44]. However, any proposed definitions of ACLF may only be regarded as transitory due to geographical constraints in both the East and the West. The various definitions have also explained significant uncertainty amongst multidisciplinary teams caring for ACLF patients. Worldwide sequentially gathered and proven data can support conventional clinical care, making a global ACLF helpful definition internationally. To collect data to develop a thorough description of ACLF, patients with extrahepatic organ failures, chronic liver disease - with or without cirrhosis, and any triggering event - intrahepatic or extrahepatic should be included. Additional work is needed to create and validate more precise prognostic ratings for various global aetiologies and phenotypes. Future research is also eagerly anticipated to address the unfulfilled medical need to detect patients who, upon admission, have a high probability of progressing to ACLF (pre-ACLF). Cutting-edge transcriptomics, proteomics, metabolomics, and biomarkers could pinpoint pre-ACLF patients, enhance medical prediction models, and give new therapeutic options [45]. An animal model of ACLF based on porcine serum-induced liver cirrhosis showed immune-metabolism dysfunction as the major process in clinical pathophysiology, thus helping explain disease etiology [46]. As prospective treatments for ACLF, several strategies focusing on immunological modulation and metabolic balance are being investigated. Glucocorticoids have been used to treat ACLF because they decrease exaggerated immune responses. However, only a tiny percentage of ACLF patients will benefit from glucocorticoids due to their adverse effects and the patients' quick alterations in immunological status. As a result, it is challenging to decide when glucocorticoids are best administered to patients with ACLF. It has been investigated whether granulocyte-colony stimulating factor, which can cause bone mesenchymal stem cell mobilization, can lower the short-term mortality of ACLF [47].

Discussion

In Asian countries, hepatitis virus infection, especially HBV, causes liver failure; in Western countries, non-alcoholic steatohepatitis and alcohol intake do [3]. Olson et al. reviewed ACLF's concept and history but only looked at current prognostic scoring methods. There is no optimum technique for quick detection of cirrhosis patients at risk of ACLF, the analysis found [12]. Guidelines regarding several aspects of ACLF that are pertinent to patterns of disease and clinical care in the Asia-Pacific area were developed in 2008 by the Asian Pacific Association for the Study of the Liver (APASL) [3]. The term 'acute deterioration in liver function over two to four weeks leading to severe deterioration in clinical status with a high Sequential Organ Failure Assessment (SOFA)/Acute Physiology and Chronic Health Evaluation (APACHE) II score with jaundice and either HE or renal failure' was proposed by Jalan et al. [4]. It has also been demonstrated that the CLIF-C ACLF score is a standalone indicator of course severity [22]. Numerous studies have shown that the likelihood of an ACLF patient surviving is inversely correlated with the extent of organ dysfunction or organ failure at diagnosis [1]. Garg et al. studied liver regeneration in a small cohort of ACLF patients using bone marrow stem cells driven by granulocyte colony-stimulating factor. The strategy reduced MELD and SOFA scores and prevented sepsis, hepatorenal syndrome, and HE, improving survival and liver function. Such results must be reproduced in other populations [47]. The only treatment that can save ACLF patients is transplantation of the liver; however, there is a shortage of evidence on transplantation of the liver for individuals with this condition, and it is unclear when and under what circumstances a transplant should be performed. Living donor transplantation is a desirable choice when the patient's prognosis depends on the timing of the transplant.

## Conclusions

A catastrophic event, ACLF has a very high short-term death rate. The severity and frequency of failing organs appear to be the primary variables predicting the eventual result mortality for those with ACLF, rather than the extent of liver disease as evaluated by traditional scores, even though its diagnosis is not well defined. The kidney, not the liver, is more susceptible to death prediction, suggesting that the underlying mechanisms behind this disease may differ from 'decompensation.' The EASL-CLIF consortium presented novel grouping standards, and only confirmation and examination of their findings can tell us if ACLF is a single group or includes groups that depict different categories with different prognoses. A theory similar to the predisposition, insult, response, organ dysfunction (PIRO) system particularly characterizes sepsis pathophysiology and can explain pathophysiology. LT and supportive treatment for organ failure are the mainstays of the current ACLF therapeutic approach. In the realm of hepatology, ACLF remains a significant challenge that requires further research to elucidate its pathophysiology and enable accurate intervention through spatiotemporal multi-omics.
